# Supplementary material for: Agility-based exercise training compared to traditional strength and balance training in older adults: a pilot randomized trial
Source: PeerJ. 2020 Apr 14;8:e8781. doi: 10.7717/peerj.8781 (PMC7164429; doi:10.7717/peerj.8781)
Supplement: Supplemental Information 2 [file peerj-08-8781-s002.docx]

✂------------------------------------------------------------------------------------------------------------------

Probandenidentifikationsnummer:

Agility Training für Senioren.

Case Report Form

Geburtsdatum: Gewicht:

Grösse: BMI:

Fettanteil: Dominantes Bein:

Untersuchungsdatum: Beinlänge:

**Tag 1**

1. Statisches Gleichgewicht einbeinig: 2. Gleichgewicht mit Perturbation:

| Bedingung | SLEO | | | |
| --- | --- | --- | --- | --- |
| Datei |  |  |  |  |
| Bemerkungen | Bein links/ rechts | | | |

| Bedingung | BLEO | | | |
| --- | --- | --- | --- | --- |
| Datei |  |  |  |  |
| Bemerkungen |  | | | |

| Spielbein | anterior | posterior lateral | posterior medial | |
| --- | --- | --- | --- | --- |
| Versuch 1 links |  |  |  | Uhrzeit: |
| Versuch 2 links |  |  |  | |
| Versuch 3 links |  |  |  | |
| Versuch 1 rechts |  |  |  | |
| Versuch 2 rechts |  |  |  | |
| Versuch 3 rechts |  |  |  | |

1. Y Balance Test:
2. Explosivkraft
   1. Plantar Flexoren (dominantes Bein zuerst)
      1. Isometrisch links / rechts

| Einstellungen Adapter | Links | Rechts |
| --- | --- | --- |
| Winkel [°] |  |  |
| Adapterlänge [cm] |  |  |

|  | Bemerkungen | |
| --- | --- | --- |
| Versuch 1 links |  | Uhrzeit: |
| Versuch 2 links |  | |
| Versuch 3 links |  | |
| Versuch 1 rechts |  | |
| Versuch 2 rechts |  | |
| Versuch 3 rechts |  | |

- 1. Dorsal Extensoren (dominantes Bein zuerst)
     1. Isometrisch links / rechts

|  | Bemerkungen | |
| --- | --- | --- |
| Versuch 1 links |  | Uhrzeit: |
| Versuch 2 links |  | |
| Versuch 3 links |  | |
| Versuch 1 rechts |  | |
| Versuch 2 rechts |  | |
| Versuch 3 rechts |  | |

- 1. Rumpf
     1. Isometrisch Extension / Flexion

| Einstellungen | Länge |
| --- | --- |
| Beinadapter [cm] |  |
| Schulterpolster hinten [cm] |  |
| Kopfpolster hinten [cm] |  |
| Brustpolster vorne (Schnalle bis Metall) [cm] |  |

|  | Bemerkungen | |
| --- | --- | --- |
| Versuch 1 Ext. |  | Uhrzeit: |
| Versuch 2 Ext. |  | |
| Versuch 3 Ext. |  | |
| Versuch 1 Flex. |  | |
| Versuch 2 Flex. |  | |
| Versuch 3 Flex. |  | |

- 1. Rumpf Rotation
     1. Isometrisch links / rechts

| Einstellungen Adapter | Links | Rechts |
| --- | --- | --- |
| Schulterpolster [cm] |  | |
| Hüftpolster [Stufe] |  |  |
| Hüftpolster Kurbel [cm] |  | |
| Beinpolster [Stufe] |  |  |
| Beinpolster Kurbel [cm] |  | |

|  | Bemerkungen | |
| --- | --- | --- |
| Versuch 1 links |  | Uhrzeit: |
| Versuch 2 links |  | |
| Versuch 3 links |  | |
| Versuch 1 rechts |  | |
| Versuch 2 rechts |  | |
| Versuch 3 rechts |  | |

1. 6 - Min Gehtest

|  | Bemerkungen | |
| --- | --- | --- |
| Versuch 1 | Anzahl gelaufene Stecken an 30m | Uhrzeit: |
|  |  | Total |
